# Interplay of iron and sulfur with syntrophic propionate oxidation

**DOI:** 10.3389/fmicb.2026.1798413

**Published:** 2026-04-10

**Authors:** Nils Weng, Eduardo Pinela, Sepehr Shakeri Yekta, Jan Moestedt, Maria Westerholm

**Affiliations:** 1 Department of Molecular Sciences, Swedish University of Agricultural Sciences, Uppsala, Sweden; 2 Department of Thematic Studies - Environmental Change, Linköping University, Linköping, Sweden; 3 Biogas Solutions Research Center, Linköping University, Linköping, Sweden; 4 Department of Biogas R&D, Tekniska verken i Linköping AB (publ.), Linköping, Sweden

**Keywords:** ammonia, anaerobic digestion, iron sulfate, methanogenesis, sulfide

## Abstract

In anaerobic environments, different chemical forms of iron and sulfur influence microbial community composition and functions. This study employs mesophilic and thermophilic ammonia-tolerant syntrophic propionate-oxidizing (SPO) cultures to investigate how different iron and sulfur species influence propionate oxidation, as well as downstream syntrophic acetate oxidation and methanogenesis. Elevated concentrations of both Fe^3+^ and Fe^2+^ species strongly inhibited SPO activity and suppressed propionate oxidation by the mesophilic culture. In contrast, FeSO_4_ addition to the thermophilic SPO culture markedly enhanced propionate oxidation and methane formation. Notably, neither Na_2_SO_4_ nor FeCl_2_ alone produced comparable stimulation, suggesting that the observed response was driven by a synergistic effect of Fe^2+^ and SO_4_^2−^ on the SPO microbial network. Following Fe^2+^ amendment of thermophilic cultures, a bacterium associated with the glycine cleavage pathway became enriched. Subsequently, with the onset of syntrophic propionate and acetate oxidation, the SPO candidate “*Candidatus* Thermosyntrophopropionicum ammoniitolerans,” a syntrophic acetate-oxidizing bacterium affiliated with the family Thermacetogeniaceae, and a hydrogenotrophic methanogen affiliated with the genus *Methanothermobacter* increased in relative abundance. Overall, the study demonstrates that predicting the outcomes of iron amendments to the anaerobic microbiome demands careful consideration of the prevailing iron and sulfur chemical speciation and their relative molar concentrations, as these factors drive divergent microbial responses under mesophilic and thermophilic conditions. The outcomes support developing targeted strategies to optimize anaerobic digestion and enhance renewable methane yields in high-ammonia biogas systems.

## 1 Introduction

Microbial propionate oxidation to acetate, carbon dioxide, and hydrogen is a key intermediate pathway in the conversion of organic wastes to biomethane in anaerobic digesters. Because this reaction is endergonic under standard conditions, it becomes thermodynamically feasible only at low formate concentrations or hydrogen partial pressures, necessitating syntrophic cooperation between propionate-oxidizing bacteria and hydrogen/formate-utilizing microorganisms, primarily methanogens (McInerney et al., 1979; Stams, 1994). The activity of syntrophic propionate-oxidizing bacteria (SPOB) in anaerobic digesters operating at high loading rates of feedstock can be constrained by elevated hydrogen partial pressures (Nielsen et al., 2007). The consequent propionate accumulation causes a cascade of adverse impacts on the anaerobic degradation chain including acidification and microbial toxicity (Li et al., 2020). Propionate tends to persist once accumulated and it is often hard to remedy the associated process perturbations in anaerobic digesters (Gallert and Winter, 2008; Safaric et al., 2018; Khafipour et al., 2020).

Anaerobic digestion of protein-rich organic wastes with commonly high methane potentials, such as slaughterhouse and food wastes, encounters yet another operational challenge due to the formation of ammonia and its inhibitory effects on microbial activity (Müller et al., 2016; Wang et al., 2022). This inhibition is more pronounced at higher pH and temperatures, where the equilibrium between ammonium (NH_4_^+^) and ammonia (NH_3_) shifts toward the latter, which is particularly toxic to acetoclastic methanogenesis (Astals et al., 2018; Jiang et al., 2019). Acetate oxidation can however proceed at high ammonia levels through syntrophic acetate oxidation (SAO) performed by syntrophic acetate-oxidizing bacteria (SAOB) in cooperation with hydrogenotrophic methanogens (Dyksma et al., 2020). Methane formation in such systems still relies on ammonia-tolerant SPOB, which differ taxonomically from those found in low-ammonia systems (Singh et al., 2021; Singh et al., 2023). SAO is likewise thermodynamically feasible only at low formate concentrations or hydrogen partial pressures (Shock and Helgeson, 1990), making efficient hydrogen/formate consumption a prerequisite for stable process performance and methane production during anaerobic digestion of protein-rich substrates.

Among other key factors, iron (Fe) and sulfur (S) chemical species are particularly influential for microbial functions during the anaerobic degradation of protein-rich organic wastes. Both Fe and S are essential nutrients as propionate oxidation and methanogenic pathways depend on a suite of Fe- and S-containing enzymes (Glass and Orphan, 2012; Patón et al., 2020; Weng et al., 2025). Desulfhydrase activity generates hydrogen sulfide (H_2_S and HS^−^) from the sulfur-containing amino acids cysteine and methionine (Gottschalk, 1986), which can interfere with microbial functions through mechanisms such as cellular toxicity or reactions with micronutrient metals (Shakeri Yekta et al., 2023). Hydrogen sulfide is also formed through dissimilatory sulfate reduction in the presence of sulfate, a process in which some SPOB and thermophilic SAOB are capable of using sulfate as an electron acceptor (Hattori et al., 2000; Westerholm et al., 2021). In practical settings, H_2_S present in biogas damages process equipment due to its corrosive nature. To mitigate process disturbances associated with its formation, Fe is commonly added to remove sulfide from the aqueous phase through FeS (Pudi et al., 2022). The FeS formed may subsequently be colonized by microorganisms (Pinela et al., 2024), or may enhance electron-transfer capacity due to its conductive properties (Kondo et al., 2015), potentially favoring the establishment of syntrophic associations in anaerobic systems.

Collectively, there is compelling evidence that the concentration and chemical speciation of Fe and S influence the activity of SPOB. However, it remains unclear how common Fe and S chemical species present in anaerobic digesters intervene with SPO. This study investigates the impact of Fe^3+^, Fe^2+^, sulfide (S^2−^), and sulfate (S^6+^) species prevalent in anaerobic digesters on SPO at high ammonia levels. For this purpose, mesophilic and thermophilic enrichment cultures of ammonia-tolerant SPOB were initially exposed to these Fe and S species, and microbial community structure was subsequently analyzed in cultures that responded positively to the additives, as indicated by increased propionate oxidation rates. The outcomes provide mechanistic insights into how Fe and S species influence SPO, with implications for optimizing the performance of anaerobic digesters operating under high-ammonia conditions.

## 2 Materials and methods

### 2.1 Enrichment cultures

The mesophilic propionate-oxidizing enrichment culture was obtained from laboratory bioreactors operating at 37 °C, originally inoculated with a microbial community from a mesophilic, high-ammonia anaerobic digester. The bioreactors were continuously fed with medium containing 0.2 g L^−1^ yeast extract, vitamins and trace elements, 0.5 g L^−1^ cysteine-HCl, 0.24 g L^−1^ Na_2_S.9H_2_O 9.6 g L^−1^ sodium propionate, and 16 g L^−1^ ammonium chloride (5 g NH_4_^+^-N L^−1^, 0.4–0.6 g NH_3_-N L^−1^) (Singh et al., 2021). Previous 16S rRNA gene-based microbial community analysis of the enrichment culture revealed the presence of several species, while metatranscriptomic analyses demonstrated that the species involved in SPO and syntrophic acetate oxidation were the ammonia-tolerant SPOB “*Candidatus* Syntrophopropionicum amoniitolerans” (Singh et al., 2021), the SAOB *Syntrophaceticus schinkii* (Westerholm et al., 2010), the hydrogenotrophic methanogen “*Candidatus* Methanoculleus ammoniitolerans” (Weng et al., 2024). The thermophilic propionate-oxidizing culture was sourced from laboratory bioreactors operating at 52 °C (Singh et al., 2023), originally inoculated from a thermophilic, high-ammonia anaerobic digester. The bioreactors were continuously supplied with the medium described above, containing 9.6 g L^−1^ sodium propionate and 9 g L^−1^ ammonium chloride (3 g NH_4_^+^-N L^−1^, 0.6–0.7 g NH_3_-N L^−1^). The thermophilic bioreactors operated continuously for 2 years, after which they were maintained under semi-continuous feeding (once a week) for an additional 4 years. The microbial enrichment comprised several species, of which the ammonia-tolerant SPOB “*Candidatus* Thermosyntrophopropionicum ammoniitolerans,” (Cheng et al., 2025) a novel SAOB affiliated with the family Thermacetogeniaceae, and methanogens (Weng et al., 2024) “*Candidatus* Methanoculleus thermohydrogenotrophicum” and *Methanothermobacter* sp. were shown by metatranscriptomic analysis to be actively involved in SPO and syntrophic acetate oxidation (Singh et al., 2023).

In the present study, the anaerobic medium was prepared according to Westerholm et al. (2010). Briefly, the medium was boiled (20 min), cooled and dispensed into serum bottles under continuous N_2_ flushing. Bottles were sealed, flushed with CO_2_/N_2_ (20:80), pressurized (0.1 atm), and autoclaved (121 °C, 20 min). After cooling, vitamin and mineral solutions were aseptically added, followed by reducing reagents (0.5 g L^−1^ cysteine-HCl and 0.24 g L^−1^ Na_2_S.9H_2_O). Resazurin (0.5 mg L^−1^) was included as redox indicator. The initial pH of the medium was 7.3. Prior to inoculation, the medium contained 0.01 mM FeCl_2_, 0.00014 mM Fe originating from the yeast extract, and a total sulfide concentration corresponding to 1 mM Na_2_S. The mesophilic cultures were supplemented with 4.8 g L^−1^ (47 mM) sodium propionate and 16 g L^−1^ (0.3 M) ammonium chloride, and incubated at 37 °C, while the thermophilic culture was supplemented with 3.7 g L^−1^ (39 mM) sodium propionate and 8.9 g L^−1^ (0.17 M) ammonium chloride, and incubated at 52 °C. The ammonium levels reflect the concentrations in their corresponding source bioreactor (Singh et al., 2021, 2023). The mesophilic assays were inoculated with 5% (v/v) of the enrichment culture. The thermophilic assays were inoculated with 30% (v/v) of the enrichment culture, as prior attempts with lower inoculum levels failed to initiate thermophilic SPO (Singh et al., 2023). If not stated otherwise, cultivations were conducted in 0.5 L serum bottles containing 0.25 L of medium. After inoculation, the gas-phase volume was 0.24 L in mesophilic cultures and 0.18 L in thermophilic cultures.

### 2.2 Assessing the influence of Fe and S chemical species on SPO

**Figure 1:**
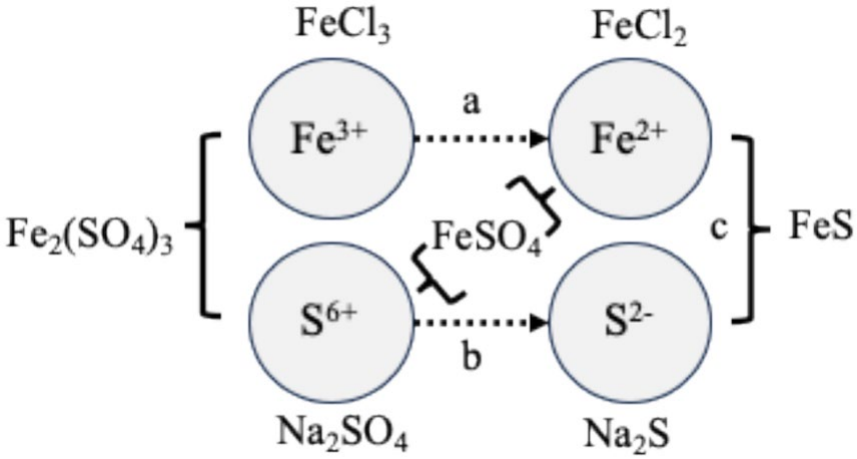
Schematic representation of the experimental set-up. FeCl_2_, FeCl_3_, Na_2_SO_4_, and Na_2_S represent excess of Fe^2+^, Fe^3+^, SO_4_^−2^, and S^2−^ ions, respectively. Fe_2_(SO_4_)_3_ and FeSO_4_ represent the co-occurrence of Fe^3+^ or Fe^2+^ with SO_4_^−2^, while FeS represents the copresence of Fe^2+^ and S^2−^. Comparison of the response of propionate-oxidizing cultures upon exposure to these additives enables assessement of the influence of major redox reactions involving Fe and S species, including (a) reduction of Fe^3+^ to Fe^2+^, (b) sulfate reduction to sulfide, and (c) precipitation of FeS.

To assess the influence of Fe and S species on SPO, the enrichment cultures were exposed to different chemical forms of these elements. Initially, a dose–response experiment was performed to evaluate the effect of Fe concentration. Under anaerobic conditions, 60 mL of mesophilic SPO culture were transferred into sterile 0.118 L serum bottles, and propionate (32 mM) and FeCl_2_ were added to obtain final concentrations of 0.1, 0.2, 0.5, 1.0, 2.0 mM. Based on these results, Fe concentration exceeding 2.0 mM were selected for subsequent experiments to ensure a measurable microbial response. The compounds FeSO_4_, FeCl_2_, Na_2_SO_4_, FeS, FeCl_3_, Fe_2_(SO_4_)_3_, Na_2_S were selected to collectively represent the major oxidation states of Fe and S in anaerobic digesters (Figure 1; Shakeri Yekta et al., 2017). To assess the effects of these species, triplicate 0.5 L serum bottles containing 0.25 L medium were supplemented with 2.3 mM FeSO_4_, FeCl_2_, Na_2_SO_4_, FeS, or FeCl_3_. Fe_2_(SO_4_)_3_ was added at 1.15 mM, while Na_2_S was added to a final concentration of 7 mM to represent highly sulfidic conditions. Triplicate control assays without additives were included.

Additional tests were conducted to analyse the influence of initial pH and to monitor H_2_S formation and H_2_ levels during growth of the thermophilic SPO culture. The initial pH was adjusted to 7.7 by reducing the KH_2_PO_4_ concentration in the medium to 0.136 g L^−1^ and KCl was added to maintain the original potassium concentration. Triplicate control assays and assays supplemented with 2.3 mM of FeSO_4_ or Na_2_SO_4_ were prepared using the modified medium. These cultivations were conducted in 0.32 L anaerobic serum bottles containing 0.12 L of the modified medium and inoculated with 30% (v/v) of the thermophilic SPO enrichment culture, providing a head space volume of 0.164 L.

### 2.3 Monitoring and analyses

The SPO activity was monitored by regular pressure readings (GMH 3100 Series, Senseca Germany GmbH). Additionally, 900 μL samples were withdrawn for analyses of acetate, propionate, butyrate, isobutyrate, valerate, isovalerate, capronate and isocapronate. Samples were prepared by pipetting 700 μL into a microcentrifuge tube, to which 70 μL (10% v/v) of H_2_SO_4_ (5 M) was added before centrifugation at 14,000 rcf for 15 min. The supernatant was then filtered into a glass vial through a 0.2 μm syringe filter. Carboxylic acids were analyzed by high-performance liquid chromatography (HPLC; Shimadzu 2050), equipped with an ion exclusion column (Rezex ROA - Organic Acid H+, 300 × 7.80 mm, Phenomenex) and a UV detector. The mobile phase was 5 mM H_2_SO_4_ with a flow rate of 0.6 mL min^−1^. The concentrations of CH_4_ and CO_2_ were regularly quantified using Clarus 500 gas chromatography equipped with a 70 HayeSep N 60/80, 1/8” SF column and an FID detector and results are reported at 25 °C and atmospheric pressure. The measurements were used to calculate propionate oxidation rates, methane production rates, and carbon balance (detailed in Supplementary Tables S1–S3). To measure H_2_ and assess if a higher initial pH promoted thermophilic SPO cultures (with and without FeSO_4_), cultivations were conducted in a medium containing lower KH_2_PO_4_ (0.136 g L^−1^). KCl was added to match the K^+^ concentration of the unmodified medium. After propionate oxidation had commenced, 2.3 mM FeSO_4_ was added to triplicate cultures. The H_2_ partial pressure was measured on two occasions in the triplicate control and FeSO_4_-amended cultures using a gas chromatograph equipped with a reducing compound photometer detector (Peak Performer 1 Reduced Gas Analyzer PP1, Peak Laboratories, CA, United States) as described previously (Westerholm et al., 2019). H_2_S concentrations were measured using a portable gas analyzer (Biogas5000, Geotech). Prior to measurement, a gas sampling bag was evacuated using a vacuum pump. Subsequently, 30–60 mL of gas from the enrichment culture was transferred into the bag using a syringe, and the remaining volume was adjusted to 1,200 mL with N_2_, corresponding to dilutions of 1:40 and 1:20, respectively. A calibration curve was constructed based on triplicate measurements of standards prepared from 1,000 ppm H_2_S standard gas. Calibration standards contained 60, 120, and 240 mL of the standard gas, with the remaining volume made up of N_2_, corresponding to H_2_S concentrations of 50, 100, and 200 ppm, respectively.

Propionate and methane turnover rates were calculated via linear regression. For propionate, regression points were selected from the interval corresponding to 15–85% of total propionate conversion, defined relative to the first and last propionate measurements, with the nearest additional time point included if fewer than three points met this criterion. For methane, regression points included all measurements corresponding to 10–90% of the final cumulative methane production. Differences in turnover rates between each additive and the control were assessed using Dunnett’s test.

### 2.4 Microbial community analyses

The assays that positively responded to the additives in terms of the rate and degree of propionate oxidation were repeated for microbial community analyses. The inoculum was obtained from the thermophilic bioreactor, but the batch assays required such large volumes that it was not possible to start all triplicate batches simultaneously. Therefore, one batch of each triplicate set was initiated 1 week apart to minimize disturbance of the bioreactor microbial community caused by large-volume withdrawal. For DNA extraction, 2 mL samples were withdrawn at three to four time points and stored at −20 °C. The DNA was extracted using the DNeasy Blood and Tissue Kit (Qiagen), and 16S rRNA gene sequencing was performed using the primers 515F/806R to amplify the V4 region. Library preparation and sequencing were carried out by Novogene Company Limited (Cambridge, UK). Amplicon sequence variants and abundance tables were inferred in R (v 4.5.1) using DADA2 (v 1.36) (Callahan et al., 2016). Forward and reverse reads were truncated to 220 bp and filtered using maximum expected error thresholds of maxEE = (2,2) and with the default value of truncQ = 2. ASVs were taxonomically assigned using a DADA2-formatted reference database derived from Silva (v 138.2) (Callahan, 2024; Chuvochina et al., 2025). Subsequent data processing and visualization were performed using phyloseq (v1.52) (McMurdie and Holmes, 2013). For visualization of the results, triplicate samples collected within 3-day intervals were grouped into the same week to account for variations in sampling time. Representative sequences of the ~250 bp long 16S rRNA gene fragments were submitted to the nucleotide BLAST database of the National Center for Biotechnology Information using parameters that excluded model organisms and uncultured/environmental sample sequences to identify closely related species based on 16S rRNA gene similarity.

Quantitative PCR (qPCR) was performed to determine the 16S rRNA gene copy number of methanogens. The primer pairs MMBf (5′-ATCGRTACGGGTTGTGGG-3′), MMBr (5′-CACCTAACGCRCATHGTTTAC-3′) and MBTf (5′-CGWAG GGAAG CTGTT AAGT-3′), MBTr (5′-TACCG TCGTC CACTC CTT-3′) were used to determine the 16S rRNA gene levels of methanogens of the order Methanomicrobiales and Methanobacteriales, respectively (Yu et al., 2005). The purified DNA extracts were diluted 10-fold prior to qPCR analysis. The qPCR protocol was as follows: 7 min at 95 °C, 40 cycles of 95 °C for 40 s, annealing at 66 or 61 °C (for the order Methanomicrobiales and Methanobacteriales, respectively) for 1 min and 72 °C for 40 s, and melting curve analysis at 95 °C for 15 s, followed by 1 min at 55 °C and finally at 95 °C for 1 s. The reactions were carried out using QuantStudio™ 5 (ThermoFisher).

### 2.5 Metagenomic analyses

Previously obtained metagenomes of active members of the SPO cultures were used to analyse the presence of iron-related genes using the FeGenie database (Garber et al., 2020). The metagenomes available at NCBI repository https://www.ncbi.nlm.nih.gov/ from the mesophilic SPO culture (Weng et al., 2024) include BioProject PRJNA1016301, “*Ca.* S. ammoniitolerans” MAG15 JAVTVF010000000, “*Ca.* M. ammoniitolerans” MAG17 JAVTVH010000000, *S. schinkii* MAG18 JAVTVI010000000 *Acetomicrobium* sp. MAG16 JAVTVG000000000. For the thermophilic SPO culture (Singh et al., 2023) BioProject PRJNA944114, the SPOB candidate “*Ca*. T. ammoniitolerans” MAG4, ASM3158407v1, the SAOB candidate *Thermacetogeniaceae* sp. MAG9 ASM3158403v1, the methanogen “*Ca*. M. thermohydrogenotrophicum” MAG1 ASM3158409v1, and *Acetomicrobium* sp. MAG5 ASM3158405v1 were analyzed.

## 3 Results

### 3.1 Syntrophic propionate oxidation by enrichment cultures

**Figure 2:**
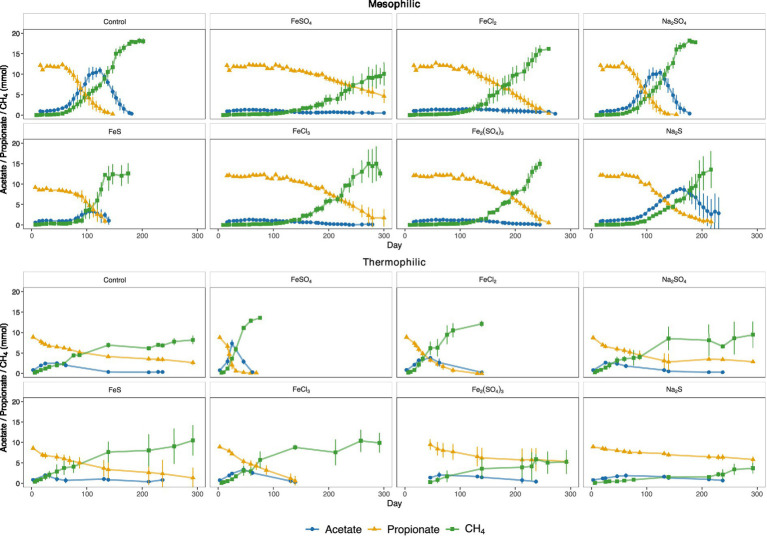
Propionate and acetate oxidation and methane production in the mesophilic (top) and thermophilic (bottom) enrichment assays amended with different Fe and S species. The error bars represent the standard deviation of triplicate batch assays.

In the mesophilic SPO cultures, additions of 0.1 and 0.2 mM FeCl_2_ showed no observable effect, whereas higher concentrations (0.5–2.0 mM FeCl_2_) led to reduced propionate oxidation rates (Figure 2; Supplementary Figure S1). In the mesophilic control cultures without additives, the propionate oxidation and methane production rates reached 0.20 and 0.16 mmol day^−1^, respectively (Supplementary Tables S1, S2). All additives, except Na_2_SO_4_, significantly (*p* < 0.05) reduced the mesophilic SPO rates compared to the control (Supplementary Table S1; Supplementary Figure S2). The mesophilic control assays required 158 days to completely degrade 47 mM propionate, and the formed acetate from SPO was degraded within 200 days. In the control and the Na_2_SO_4_, Na_2_S and FeS treatments, acetate accumulated to 10–15 mM, indicating that the SAO community could degrade acetate as rapidly as it was produced. In contrast, treatments with lower SPO rates did not show acetate accumulation. The treatments that achieved complete oxidation of propionate and acetate within the 270-day experimental period (FeCl_2_, Na_2_SO_4_, FeS and Fe_2_(SO_4_)_3_, Figure 2), yielded methane production comparable to the control, corresponding to 83–91% of the theoretical maximum (Supplementary Table S2).

Under thermophilic conditions, propionate oxidation proceeded very slowly in the control cultures and in the cultures amended with Na_2_SO_4_, FeS, Fe_2_(SO_4_)_3_ and Na_2_S, with approximately 35 mM propionate remaining at the end of the incubation period (Figure 2). In contrast, supplementation with FeSO_4_ and FeCl_2_ significantly enhanced the thermophilic propionate oxidation and methane generation rates compared with the control (Figure 2, Supplementary Tables S1, S2). However, the stimulatory effect of FeCl_2_ on SPO was not reproducible, as no enhancement was observed when the experiment was repeated to evaluate microbial community responses in the thermophilic enrichment culture (Supplementary Figures S3, S4; Supplementary Table S3). The amendment with FeCl_3_ also significantly increased propionate oxidation rates relative to the control, although this effect was less pronounced than that observed with FeSO_4_ and FeCl_2_ (Supplementary Table S1). Carbon balance calculations revealed that, on average, 71–78% of the propionate-derived carbon was recovered as methane in the control and FeCl_2_- or FeCl_3_-amended cultures. In contrast, methane recovery in the FeSO_4_ cultures reached approximately 90% of the theoretical maximum, while FeS treatments showed 82% recovery and Fe_2_(SO_4_)_3_ and Na_2_S treatments showed 68–69% recovery (Supplementary Table S2). The H_2_ partial pressure decreased from 72 to 23 Pa in the control batches, from 70 to 25 Pa in the NaSO_4_-amended batches and varied between 79 and 168 Pa in the FeSO_4_-amended batches, during a period in which propionate was degraded. The H_2_S levels were about 5,900–6,300 ppm in control and NaSO_4_-amended cultures and increased from 700 ppm after 7 days of incubation to 5,100 ppm after 17 days of incubation in cultures with FeSO_4_ (Supplementary Table S4).

### 3.2 Microbial community analyses and iron-related genes

**Figure 3:**
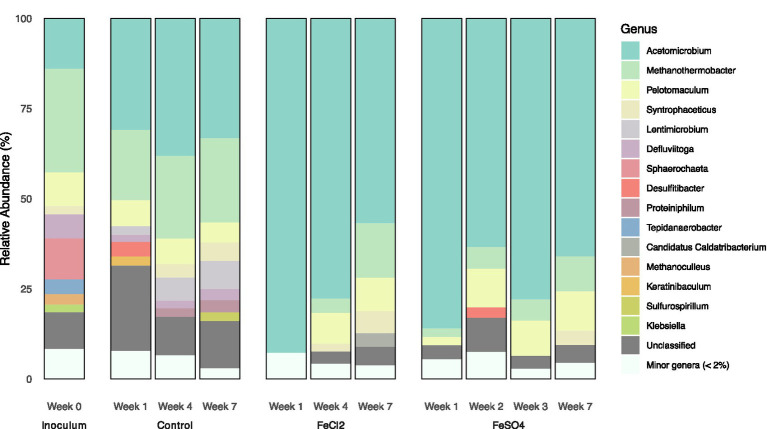
Microbial community composition at the genus level in thermophilic cultures, including the inoculum, control, and assays supplemented with FeCl_2_ and FeSO_4_. Merged sequence data from triplicate cultures are shown. Minor genera include taxa with relative abundance below 2% and ASVs lacking taxonomic assignment at genus level are grouped as “Unclassified.” Samples for community analyses were collected at weeks 1, 4, and 7 in the control and FeCl_2_-amended batches, and at weeks 1, 2, 3 (during exponential propionate oxidation), and 7 (during acetate oxidation) in the FeSO_4_-amended assays. The 16S rRNA gene sequences affiliated with the genus *Pelotomaculum* showed 100% sequence similarity to the thermophilic SPOB candidate “*Ca.* T. ammoniitolerans.”

The microbial community analysis of the thermophilic SPO assays demonstrated that species within the genera of *Methanothermobacter Pelotomaculum*, *Syntrophaceticus* and *Acetomicrobium* were present at high relative abundance in the inoculum taken from the bioreactor and the batch assays. The BLAST search and gene comparison with previously obtained MAGs from the enrichment culture described in section 2.5 demonstrated taxonomic similarity with the hydrogenotrophic *Methanothermobacter tenebrarum* (100% sequence similarity) (Nakamura et al., 2013), SPOB candidate “Ca. T. ammoniitolerans” (100% sequence similarity, assigned as *Pelotomaculum* in Figure 3) and previously identified thermophilic SAOB candidate (100%, assigned as *Syntrophaceticus* in Figure 3) (Singh et al., 2023). Based on BLAST analysis, the closest relative of the *Acetomicrobium* sp. was *Acetomicrobium mobile*, sharing 98.4% sequence similarity. This species has been shown to ferment sugars and yeast extract to hydrogen, CO_2_ and acetate, and to reduce cysteine to sulfide using glucose or compounds in yeast extract as electron donors, but it is incapable of sulfate reduction (Menes and Muxí, 2002; Hania et al., 2016).

In the thermophilic inoculum, *Methanothermobacter* dominated the community, representing 29% of sequences, followed by *Acetomicrobium* (14%) and “*Ca.* T. ammoniitolerans” (9%, Figure 3). During incubation without amendment, where SPO activity remained low, the community composition was largely stable over time, except for an increase in the relative abundances of the putative SAOB (from 1 to 5%) and *Lentimicrobium* (from 2 to 8%). In the FeSO_4_ and Fe_2_Cl amended assays, *Acetomicrobium* initially dominated the communities, representing 86–93% at week 1. During incubation, the relative abundances of *Methanothermobacter* increased from 1.8–2.4% to 10–15%, “*Ca. T. ammoniitolerans*” from 2 to 11%, and the putative SAOB from below 0.3–0.4 to 6% in both FeCl- and FeSO_4_-amended cultures. The main difference between the amended cultures was the higher relative abundance of both the SPOB and *Methanothermobacter* at the first sampling point in the FeSO_4_-amended cultures.

The FeCl_2_- and FeSO_4_-amended cultures showed pronounced community shifts already within the first week, with *Acetomicrobium* comprising approximately 90% of total sequences. Over the course of the experiment, the relative abundance of the SPOB candidate “*Ca.* T. ammoniitolerans” increased to 10–12% and *Methanothermobacter* to 11–15% in both treatments. By week 7 in the FeSO_4_-amended cultures, when acetate oxidation had commenced, the relative abundance of the SAOB candidate had increased to 3.3–6.5% (Figure 3). qPCR analysis showed that Methanobacteriales were consistently more abundant than Methanomicrobiales and remained stable over time, matching the higher abundance of *Methanothermobacter* (belonging to Methanobacteriales) relative to *Methanoculleus* (belonging to Methanomicrobiales) observed in Figure 3 (Supplementary Figure S5). The MAGs of both the mesophilic and thermophilic SPOB, SAOB, the methanogens and *Acetomicrobium* encoded the Fe^2+^ transporters *feoA* and *feoB* but lacked the regulator *feoC* (Lau et al., 2015). The mesophilic methanogen encoded ATP-binding cassette (ABC) transporters of iron-containing compounds. Among the species examined, *Acetomicrobium* uniquely possessed genes encoding an ABC-type Fe^3+^ transport system (*fbp*ABC) and proteins involved in siderophore-mediated iron acquisition (*Exb*BD, *Ton*B). None of the MAGs harbored genes for iron reduction or iron oxidation (Supplementary Figure S6).

## 4 Discussion

### 4.1 Inhibition of mesophilic SPO by Fe

The negative effects of FeCl_2_, FeSO_4_, FeCl_3_ and Fe_2_(SO_4_)_3_ additions on mesophilic SPO are likely attributable to a combination of chemical and biological factors. One possible explanation is that the excess dissolved Fe^2+^ or Fe^3+^ in the anaerobic medium scavenges sulfide, thereby reducing sulfur availability for essential cellular functions, such as assimilation by methanogens (Rönnow and Gunnarsson, 1981; Perona et al., 2018). However, given the high molar sulfate concentrations in the FeSO_4_- and Fe_2_(SO_4_)_3_-amended assays and the presence of Na_2_S in the medium, sulfur limitation is unlikely to explain the reduced activity observed in the mesophilic SPO culture. Furthermore, cysteine supplied as a reducing agent in the present medium may serve as an additional S source (Li et al., 2020). Earlier metagenomic analyses of the same culture including “*Ca.* S. ammoniitolerans”, *S. schinkii* and “*Ca.* M. ammoniitolerans” identified amino acid sequences annotated to cysteine biosynthesis and transport (e.g., Cysteine tRNA ligase) as well as sulfate permease (e.g., YeiH family putative sulfate export transporter) (Weng et al., 2024). Consequently, sulfur acquisition by the mesophilic SPO culture via either assimilatory sulfate reduction or amino acid uptake cannot be excluded based on available information. In addition, the absence of any response in propionate oxidation rate to Na_2_SO_4_ supplementation and the negative effect of FeSO_4_ supplementation suggest that the mesophilic SPOB lack the capacity for dissimilatory sulfate reduction using propionate as an electron donor. This interpretation is consistent with previous metagenomic analyses of the mesophilic SPOB candidate, which indicated absence of genes required for sulfate reduction (Singh et al., 2021). Similarly, sulfate reduction has not been observed in pure cultures of the mesophilic SAOB *S. schinkii* (Westerholm et al., 2010), further supporting the conclusion that these organisms do not possess this metabolic capability. The results further indicate that the mesophilic SPOB, the SAOB and the methanogen were relatively tolerant to elevated hydrogen sulfide concentrations, as Na_2_S supplementation caused less inhibition than Fe_2_(SO_4_)_3_ or FeSO_4_ supplementation.

Overall, the results indicate that the inhibitory effects on SPO under mesophilic conditions were primarily associated with Fe^2+^ and Fe^3+^ ions rather than the accompanying anions or reduced sulfur availability. Although reduction of Fe^3+^ supplied as Fe_2_(SO_4_)_3_ and FeCl_3_ could transiently increase the redox potential and potentially inhibit SPO, the comparable inhibition observed following Fe^2+^ and Fe^3+^ amendments indicates that the redox effect was not a decisive factor controlling SPO or methanogenesis. In anaerobic microbial systems, adverse effects of excess Fe^3+^ have been attributed to competition between Fe^3+^-reducing bacteria and methanogens for acetate and hydrogen, methanogenic Fe^3+^ reduction, or direct inhibition by Fe^3+^ ions (Bodegom et al., 2004). While some iron-reducing bacteria (e.g., *Geobacter* spp.) can couple propionate oxidize to iron reduction (Lovley et al., 1993), such activity was evidently absent in the present mesophilic enrichments. Although the propionate oxidation rate was slower, carbon balance calculations showed that total methane yields were comparable to the control, effectively ruling out the presence of competing microbial Fe^3+^-reducing pathways in the mesophilic culture (Supplementary Tables S1, S2).

Taken together, our results point to a direct toxic effect of iron on key microorganisms involved in SPO under mesophilic and high-ammonia conditions. Inhibition of the methanogens in pure cultures has been demonstrated previously, showing that methanogens grown on H_2_/CO_2_ were particularly sensitive to Fe^3+^, with inhibition observed at concentrations as low as 1 mM, which was suggested to be due to Fe^3+^ adsorption to outer-membrane cofactors and proteins (Bodegom et al., 2004; Zhang et al., 2009). Notably, in mesophilic high-ammonia biogas digesters, trace element supplementation including Fe^2+^ and Fe^3+^, has been shown to decrease the abundance of the SAOB *S. schinkii*, while concomitantly increasing *Methanosarcina* populations (Westerholm et al., 2015), indicating that iron additions can favor the acetoclastic pathway over SAO in mesophilic and high-ammonia conditions. Although several studies have reported reduced propionate accumulation in biogas systems following supplementation with iron salts containing FeCl_3_ (Schmidt et al., 2014) or FeCl_2_ (Osuna et al., 2003; Capson-Tojo et al., 2018; Zhang et al., 2019; Wang et al., 2020), these effects are often confounded by co-addition of other trace elements or by interactions between iron and sulfur compounds. Moreover, given the highly interconnected nature of anaerobic degradation pathways, it is difficult to discern whether iron addition directly impacts propionate oxidation or instead altered upstream or parallel pathways, resulting in reduced propionate formation.

The inhibition by iron observed in the present study was unexpected, given earlier findings with the same enrichment culture showing that supplementation with iron oxides (Fe_3_O_4_) enhanced mesophilic SPO activity. In that study, Fe_3_O_4_ was added as nanoparticles that were subsequently transformed into iron sulfide (mainly FeS_2_ and FeS) and became colonized by microorganisms (Pinela et al., 2024). A similar stimulatory effect might therefore have been expected following FeS addition. However, no enhancement of SPO was observed under those conditions. Together, these observations suggest that iron supplied as poorly soluble, particulate forms with limited bioavailability, has neutral or mildly positive effects, whereas iron added in more soluble forms can inhibit mesophilic SPO. The interpretation is also supported by a previous study of the same mesophilic SPO culture cultivated under static and mechanically mixed conditions, in which both the SPOB and the methanogen downregulated ferrous iron transport proteins during mixing (Weng et al., 2025). Considering the insights from the present study, it is plausible that improved diffusion at the cell surface under the mixing conditions in the previous study may have increased iron exposure at the cell surface, leading to intracellular iron levels that were inhibitory rather than beneficial.

### 4.2 Slow rate of thermophilic SPO without excess Fe and S

The thermophilic SPO culture without excess Fe and S exhibited markedly slower propionate oxidation (0.02 mmol day^−1^) than the mesophilic culture (0.2 mmol day^−1^). The slow oxidation under thermophilic conditions was hypothesized to relate to the lower starting pH of the medium (7.3) relative to the source bioreactor (~8.0). However, increasing the initial pH to 7.7 only slightly enhanced SPO rates as compared to the controls starting at a pH of 7.3 (during the first 20 days of cultivation, Supplementary Tables S1, S3, S4). The rate of thermophilic SPO was also substantially lower than previously reported for cultures derived from the same inoculum source (Singh et al., 2023). In that study, cultures with limited SPO activity were associated with a low abundance of hydrogenotrophic *Methanothermobacter* and dominance of *Methanoculleus*. In contrast, in the present study *Methanothermobacter* was more abundant than *Methanoculleus* (Figure 3; Supplementary Figure S5). The co-existence of hydrogenotrophic methanogens with complementary electron donor preferences has been proposed to support efficient SPO. For example, McDaniel et al. (2023) reported that two *Methanothermobacter* species were involved in hydrogen and formate consumption, respectively thereby supporting SAO in a thermophilic culture. The dominance of *Methanothermobacter* and the lower abundance of *Methanoculleus* observed here may therefore have reduced functional complementarity within the methanogenic community, potentially contributing to the low SPO rates observed in the control assays.

### 4.3 The role of *Acetomicrobium* sp.

**Figure 4:**
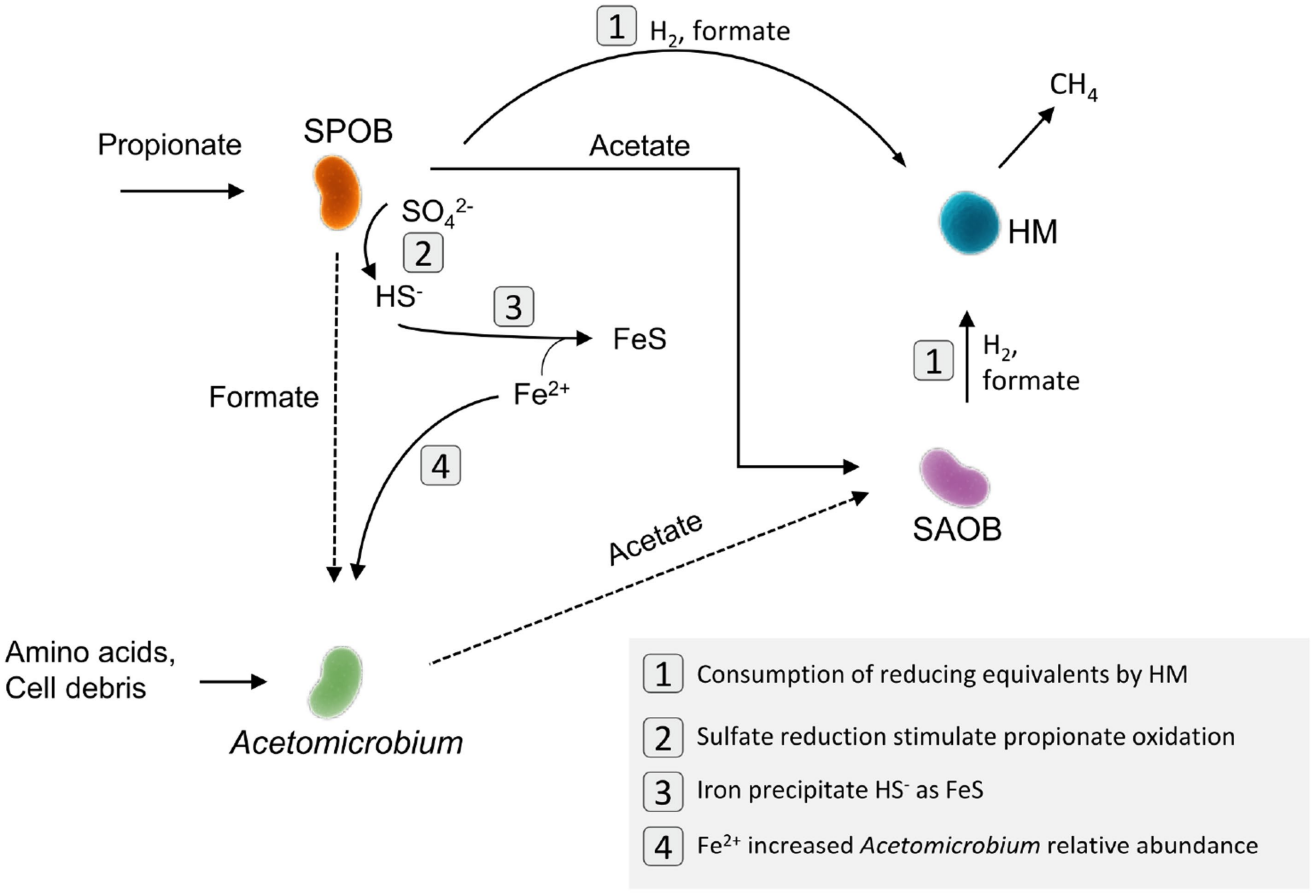
Putative intervention pathways of FeSO_4_ in syntrophic propionate oxidation in the thermophilic culture. The picture depicts mechanisms that potentially (but not exclusively) stimulate syntrophic propionate oxidation and methanogenesis. In this context, Fe^2+^ increase the relative abundance of *Acetomicrobium*, while sulfate reduction stimulates the initial growth of “*Ca.* T. ammoniitolerans” (the candidate syntrophic propionate-oxidizing bacterium, SPOB). HM represents the hydrogenotrophic methanogens and SAOB the syntrophic acetate-oxidizing bacterium. Additional Fe^2+^ also interacts with the formed HS^−^ and precipitates as FeS.

Although propionate-oxidizing activity remained low in both the control and FeCl_2_-supplemented batch assays, the microbial community structures differed markedly (Figure 3). In the FeCl_2_-supplemented assays, a pronounced increase in the relative abundance of *Acetomicrobium* was observed, mirroring the pattern seen in FeSO_4_-supplemented cultures. This suggests that *Acetomicrobium* growth substantially benefited from increased Fe^2+^ availability (Figure 4, reaction 4). *Acetomicrobium* species have been previously identified as active members of the mesophilic and thermophilic SPO enrichment cultures examined in this study, where they were shown to lack genes encoding key enzymes in the Wood-Ljungdahl pathway but to express genes associated with the reductive glycine pathway, including the glycine cleavage system, glycine reductase complex, pyruvate synthase, and related proteins (Singh et al., 2023; Weng et al., 2025). *Acetomicrobium* sp. was shown to have a broad metabolic versatility and long-term persistent activity in the syntrophic cultures (Singh et al., 2023; Weng et al., 2025). This indicates that this species can switch between a broad range of substrate utilization pathways, using either CO_2_, formate, acetate or amino acids, which has also been proposed for other species having a similar gene expression profile (Sánchez-Andrea et al., 2020; Friedline et al., 2025). Metagenomic analyses of *Acetomicrobium* sp. identified genes encoding iron transport proteins, including chaperons involved in delivering iron required for synthesis of metalloenzymes (Supplementary Figure S6). These findings indicate that iron acquisition is important during cell division in this species (Figure 4). Furthermore, the species possessed genes encoding ferritin-like molecules that has been suggested to be used to detoxify Fe^2+^ and enable iron storage, thereby contributing to resistance under iron stress (Bennett and Gralnick, 2019).

### 4.4 Stimulation of the thermophilic SPO by FeSO_4_ and the microbial network

The FeSO_4_ addition consistently stimulated thermophilic SPO activity (Figure 2, Supplementary Figures S2, S3; Supplementary Tables S1, S3, S4). This stimulatory effect may be attributed to FeSO_4_ serving as a source of iron and sulfur required for microbial growth, or to the activation of microbial sulfate reduction. One possible explanation is hydrogen-dependent sulfate reduction by members of the enrichment community, which could decrease the hydrogen partial pressure and thereby thermodynamically stimulate SPOB activity. However, this mechanism is contradicted by the observation that hydrogen partial pressure was considerably higher in the FeSO_4_-amended assays than in the controls (Supplementary Table S4). An alternative explanation is that FeSO_4_ amendment enhanced formate consumption, potentially by stimulating *Acetomicrobium* sp. Enhanced formate consumption could alleviate formate accumulation and associated inhibition resulting from relatively slow formate utilization by methanogens in non-amended assays (Figure 4, reaction 1). This, in turn, may facilitate increased propionate oxidation rate by the SPOB. Previous gene expression analyses suggest that the thermophilic syntrophic microorganisms investigated here are capable of using both molecular hydrogen and formate as electron carriers for interspecies electron transfer (Singh et al., 2023; Weng et al., 2024). However, any effects on formate turnover await confirmation through further targeted analyses.

A more plausible explanation for the increased propionate oxidation rate in the FeSO_4_-amended assays is that low levels of sulfate initially enabled partial propionate oxidation by the SPOB via sulfate reduction (Figure 4, reaction 2). This pathway may have provided a thermodynamically favorable energy yield that supported initial growth and activation the SPOB population. Once established, these microorganisms could subsequently revert to syntrophic cooperation with methanogenic partners. Indeed, sulfate reduction is well known to offer a more energetically favorable route for propionate oxidation compared to strictly syntrophic metabolism (Stams et al., 2005). This hypothesis is further supported by metagenomic evidence showing that “*Ca.* T. ammoniitolerans” encodes key components of the dissimilatory sulfate reduction pathway dissimilatory sulfite reductase (dsrC), anaerobic sulfite reductase (asrAB), adenylylsulfate reductase (aprAB), sulfate adenylyltransferase (sat), pyrophosphatase (ppaX) and ABC-type sulfate transporter (Singh et al., 2023). Notebly, sulfide introduced via 2.3 mM FeSO_4_ did not appear to negatively affect methane production. Methane yields reached up to 90% of the theoretical potential from propionate (Figure 2; Supplementary Table S2), despite the expectation that competition for reducing equivalents between sulfate reducers and methanogens would decrease methane yield. This methane yield exceeds previously reported values for mesophilic SPO, where approximately 75–78% of the carbon was recovered as methane (Pinela et al., 2024), and for mesophilic butyrate oxidation, where methane recoveries around ~80% have been reported (Ziels et al., 2019).

Furthermore, the absence of comparable response in the thermophilic SPO culture amended with Na_2_SO_4_ or Fe_2_(SO_4_)_3_ indicates that sulfate alone, or sulfate in combination with Fe^3+^, cannot explain the observed stimulation of SPO activity. Addition of Na_2_S to the thermophilic culture resulted in a lower degree of propionate conversion to methane compared to the control (Supplementary Table S2), indicating potential inhibition of the thermophilic methanogens by elevated H_2_S levels. Together with the marked decrease in H_2_S following FeSO_4_ addition, this suggests that stochiometric Fe^2+^ and SO_4_^2−^ supplied as FeSO_4_ reduced H_2_S concentrations generated during sulfate reduction via FeS precipitation, thereby alleviating H_2_S-related inhibition (Figure 4, reaction 3). A similar increase in propionate oxidation rate was not observed in the thermophilic assays amended with Fe^3+^ and SO_4_^2−^ supplied as Fe_2_(SO_4_)_3_ (Figure 2). In these assays, the molar concentration of SO_4_^2−^ is higher than Fe^3+^, and sulphidic conditions may have persisted despite FeS formation, thereby inhibiting the methanogens.

## 5 Concluding remarks

The present study investigates how distinct iron and sulfur species interact to regulate propionate oxidation, as well as downstream syntrophic acetate oxidation and methanogenesis, under both mesophilic and thermophilic conditions at moderate to high ammonia levels. In the mesophilic SPO cultures, elevated concentrations of both Fe^3+^ and Fe^2+^ strongly inhibited SPO activity. Taken together with previous results, these findings indicate that iron supplied as poorly soluble, particulate phases with limited bioavailability has neutral or mildly positive effects, whereas iron added in more soluble forms can inhibit SPO under mesophilic conditions.

In thermophilic SPO enrichment cultures, the addition of low level of FeSO_4_ (2.3 mM) consistently enhanced SPO. This indicates that, under thermophilic and high-ammonia anaerobic digesters, addition of low sulfate concentrations can transiently stimulate the onset of propionate oxidation by providing an energetically favorable sulfate-reducing pathway prior to the establishment of syntrophic cooperation. Furthermore, the results indicate that stoichiometrically balanced Fe^2+^ and SO_4_^2−^ may alleviate sulfide inhibition through FeS precipitation, thereby creating more favorable redox and toxicity conditions for thermophilic SPO under high-ammonia conditions. Furthermore, the findings point to a potential role of a glycine cleavage pathway-utilizing species within the microbial network, whose relative abundance appears to be influenced by Fe^2+^ availability. Future work should analyse sulfate reduction activity in the thermophilic SPO communities to disentangle the mechanistic role of FeSO_4_ and to assess its applicability as a targeted strategy for stabilizing thermophilic anaerobic digestion systems operating at elevated ammonia levels.

## Data Availability

The datasets presented in this study can be found in online repositories. The names of the repository/repositories and accession number(s) can be found at: https://www.ncbi.nlm.nih.gov/, BioProject PRJNA1415707.
