# Supplementary material for: Interplay of iron and sulfur with syntrophic propionate oxidation
Source: Front Microbiol. 2026 Apr 10;17:1798413. doi: 10.3389/fmicb.2026.1798413 (PMC13106459; doi:10.3389/fmicb.2026.1798413)
Supplement: Supplementary file 1 [file Data_Sheet_1.docx]

Supplementary Material

Interplay of iron and sulfur speciation with syntrophic propionate oxidation

# Supplementary Figures and Tables

## Supplementary Figures

**Figure S1**. Propionate (A) and acetate (B) degradation curves in mesophilic SPO cultures with the addition of different FeCl_2_ concentrations. 60 ml of culture that previously had degraded propionate were used to minimize lag phases in this test. Propionate was added to a final concentration of 32 mM (2.4 g L^-1^) and the cultures were supplemented with 0 (control), 0.1 (0.013 g L^-1^), 0.2 (0.025 g L^-1^), 0.5 (0.064 g L^-1^), 1.0 (0.13 g L^-1^), and 2.0 mM (0.25 g L^-1^) FeCl_2_. Triplicate assays were incubated at 37 °C. The SPO activity was monitored as detailed in the main text.

**Figure S2**. Propionate, acetate, and methane turnover in mesophilic (top) and thermophilic (bottom) enrichment assays amended with different Fe and S species; each panel represents an individual replicate. Propionate (red) and methane (blue) rates were calculated by linear regression over the 15–85% conversion window (with one additional point for thermophilic FeSO_4_ when needed) and the 10–90% final CH_4_ interval, respectively.

**Figure S3**. Propionate oxidation and acetate formation and oxidation by the thermophilic ammonia-tolerant syntrophic propionate-oxidizing culture used for microbial community analysis.

**Figure S4**. Propionate and acetate turnover in thermophilic enrichment assays used for microbial community analysis, under FeCl_2_ and FeSO_4_ amended conditions. Each panel represents an individual experimental replicate. Linear regression fits used to calculate propionate oxidation rates are shown in red. Rates were calculated over the 10–90% propionate conversion window.

**Figure S5**. Quantification of 16S rRNA gene copy numbers based on quantitative PCR with primers targeting the methanogenic orders Methanobacteriales (red) and Methanomicrobiales (blue); each panel represents an individual experimental replicate. The y axis shows the log_10_ transformed 16S rRNA gene copy number per nanogram of total DNA.

**Figure S6.** Heatmap of identified iron-related genes using the bioinformatical tool FeGenie. Rows show genes grouped by category. Column shows different genomes from mesophilic and thermophilic cultures respectively. Cell colors reflect the row-scaled counts within each temperature block (raw values shown in cells).

## Supplementary Tables

**Table 1.** Propionate oxidation rates for the mesophilic and thermophilic enrichment cultures.

| Enrichment culture | Additive | Replicate | Start and end day | Rate^1^ (mmol day^-1^) | n (regression points) | Average rate (mmol day^-1^) | Dunnett p-value^2^ |
| --- | --- | --- | --- | --- | --- | --- | --- |
| Mesophilic | Control | 1 | 83-124 | 0.17 | 7 | 0.20 ± 0.03 |  |
|  |  | 2 | 69-104 | 0.22 | 6 |  |  |
|  |  | 3 | 76-111 | 0.20 | 6 |  |  |
|  | Fe_2_(SO_4_)_3_ | 1 | 153-237 | 0.08 | 13 | 0.09 ± 0.01 | 0.000 |
|  |  | 2 | 138-237 | 0.08 | 15 |  |  |
|  |  | 3 | 147-223 | 0.09 | 12 |  |  |
|  | FeCl_2_ | 1 | 117-216 | 0.08 | 15 | 0.08 ± 0.00 | 0.000 |
|  |  | 2 | 138-230 | 0.08 | 14 |  |  |
|  |  | 3 | 132-237 | 0.07 | 16 |  |  |
|  | FeCl_3_ | 1 | 167-323 | 0.05 | 18 | 0.07 ± 0.01 | 0.000 |
|  |  | 2 | 153-260 | 0.07 | 15 |  |  |
|  |  | 3 | 153-260 | 0.07 | 15 |  |  |
|  | FeS | 1 | 70-105 | 0.14 | 6 | 0.13 ± 0.01 | 0.001 |
|  |  | 2 | 84-126 | 0.12 | 6 |  |  |
|  |  | 3 | 80-119 | 0.13 | 7 |  |  |
|  | FeSO_4_ | 1 | 138-336 | 0.03 | 23 | 0.04 ± 0.01 | 0.000 |
|  |  | 2 | 153-307 | 0.05 | 19 |  |  |
|  |  | 3 | 177-369 | 0.04 | 22 |  |  |
|  | Na_2_S | 1 | 97-177 | 0.11 | 11 | 0.11 ± 0.01 | 0.000 |
|  |  | 2 | 89-167 | 0.11 | 12 |  |  |
|  |  | 3 | 97-188 | 0.10 | 14 |  |  |
|  | Na_2_SO_4_ | 1 | 69-104 | 0.23 | 6 | 0.21 ± 0.03 | 0.963 |
|  |  | 2 | 83-124 | 0.17 | 7 |  |  |
|  |  | 3 | 83-117 | 0.21 | 6 |  |  |
| Thermophilic | Control | 1 | 17-139 | 0.03 | 9 | 0.02 ± 0.00 |  |
|  |  | 2 | 17-229 | 0.02 | 11 |  |  |
|  |  | 3 | 20-212 | 0.02 | 8 |  |  |
|  | Fe_2_(SO_4_)_3_ | 1 | 59-139 | 0.03 | 5 | 0.02 ± 0.01 | 1.000 |
|  |  | 2 | 59-139 | 0.03 | 5 |  |  |
|  |  | 3 | 59-237 | 0.01 | 8 |  |  |
|  | FeCl_2_ | 1 | 17-59 | 0.12 | 6 | 0.10 ± 0.02 | 0.000 |
|  |  | 2 | 20-69 | 0.10 | 6 |  |  |
|  |  | 3 | 20-87 | 0.09 | 7 |  |  |
|  | FeCl_3_ | 1 | 25-87 | 0.08 | 5 | 0.06 ± 0.01 | 0.034 |
|  |  | 2 | 25-87 | 0.06 | 5 |  |  |
|  |  | 3 | 25-87 | 0.05 | 5 |  |  |
|  | FeS | 1 | 20-139 | 0.04 | 8 | 0.03 ± 0.01 | 0.988 |
|  |  | 2 | 20-139 | 0.02 | 8 |  |  |
|  |  | 3 | 20-139 | 0.03 | 8 |  |  |
|  | FeSO_4_ | 1 | 17-25 | 0.60 | 3 | 0.57 ± 0.03 | 0.000 |
|  |  | 2 | 17-25 | 0.54 | 3 |  |  |
|  |  | 3 | 17-25 | 0.56 | 3 |  |  |
|  | Na_2_S | 1 | 20-237 | 0.01 | 10 | 0.01 ± 0.00 | 0.746 |
|  |  | 2 | 25-237 | 0.01 | 10 |  |  |
|  |  | 3 | 46-237 | 0.01 | 9 |  |  |
|  | Na_2_SO_4_ | 1 | 20-87 | 0.05 | 7 | 0.03 ± 0.01 | 0.948 |
|  |  | 2 | 20-139 | 0.03 | 8 |  |  |
|  |  | 3 | 20-139 | 0.03 | 8 |  |  |

^1^Propionate oxidation rates were calculated via linear regression of data points between the dates reported in Start and end day columns. These were the dates where propionate depletion fell between 15% and 85% of total propionate depletion, as described by the formula: 0.15 <= (x_start_ – x) / (x_start_-x_end_) < 0.85, where x is the moles of propionate and x_start_/x_end_ is the moles of propionate at first/last propionate data point respectively.

^2^ P-values from Dunnett’s test for propionate oxidation rates (additive vs. control).

**Table 2.** Methane production rates and propionate conversion percentages for the mesophilic and thermophilic enrichment cultures.

| Enrichment culture | Additive | Replicate | Start and end day | Rate^1^ (mmol day^-1^) | n (regression points) | Average rate (mmol day^-1^) | Conversion percentage^2^ | Dunnett p-value^2^ |
| --- | --- | --- | --- | --- | --- | --- | --- | --- |
| Mesophilic | Control | 1 | 89-160 | 0.18 | 11 | 0.16 ± 0.02 | 84.9 |  |
|  |  | 2 | 76-167 | 0.14 | 14 |  | 90.9 |  |
|  |  | 3 | 76-147 | 0.16 | 11 |  | 82.9 |  |
|  | Fe_2_(SO_4_)_3_ | 1 | 147-230 | 0.10 | 12 | 0.12 ± 0.02 | 83.8 | 0.121 |
|  |  | 2 | 147-230 | 0.13 | 12 |  | 78.6 |  |
|  |  | 3 | 153-230 | 0.14 | 11 |  | 76.7 |  |
|  | FeCl_2_ | 1 | 117-223 | 0.12 | 15 | 0.12 ± 0.01 | 83.9 | 0.095 |
|  |  | 2 | 153-260 | 0.13 | 14 |  | 90.5 |  |
|  |  | 3 | 132-237 | 0.11 | 15 |  | 77.5 |  |
|  | FeCl_3_ | 1 | 153-323 | 0.08 | 21 | 0.09 ± 0.02 | 76.4 | 0.003 |
|  |  | 2 | 153-237 | 0.10 | 12 |  | 66.1 |  |
|  |  | 3 | 153-260 | 0.11 | 14 |  | 86.5 |  |
|  | FeS | 1 | 80-105 | 0.22 | 5 | 0.23 ± 0.01 | 50.9 | 0.001 |
|  |  | 2 | 98-140 | 0.25 | 6 |  | 80.8 |  |
|  |  | 3 | 98-133 | 0.24 | 5 |  | 93.7 |  |
|  | FeSO_4_ | 1 | 132-331 | 0.04 | 26 | 0.06 ± 0.02 | 64.9 | 0.000 |
|  |  | 2 | 153-307 | 0.08 | 20 |  | 79.9 |  |
|  |  | 3 | 167-377 | 0.05 | 27 |  | 65.4 |  |
|  | Na_2_S | 1 | 111-195 | 0.11 | 13 | 0.10 ± 0.03 | 71.9 | 0.007 |
|  |  | 2 | 104-188 | 0.13 | 13 |  | 81.6 |  |
|  |  | 3 | 111-331 | 0.07 | 29 |  | 77.1 |  |
|  | Na_2_SO_4_ | 1 | 76-147 | 0.17 | 11 | 0.18 ± 0.01 | 88.7 | 0.791 |
|  |  | 2 | 89-147 | 0.17 | 9 |  | 84.8 |  |
|  |  | 3 | 89-160 | 0.18 | 11 |  | 85.9 |  |
| Thermophilic | Control | 1 | 17-87 | 0.05 | 7 | 0.03 ± 0.01 | 73.0 |  |
|  |  | 2 | 25-258 | 0.03 | 11 |  | 74.5 |  |
|  |  | 3 | 25-237 | 0.03 | 9 |  | 78.5 |  |
|  | Fe_2_(SO_4_)_3_ | 1 | 76-237 | 0.01 | 5 | 0.01 ± 0.01 | 69.0 | 0.626 |
|  |  | 2 | 59-258 | 0.03 | 7 |  | 87.9 |  |
|  |  | 3 | 139-229 | 0.00 | 3 |  | 49.0 |  |
|  | FeCl_2_ | 1 | 25-59 | 0.17 | 4 | 0.14 ± 0.03 | 79.3 | 0.000 |
|  |  | 2 | 25-87 | 0.12 | 6 |  | 74.3 |  |
|  |  | 3 | 25-87 | 0.12 | 6 |  | 81.0 |  |
|  | FeCl_3_ | 1 | 25-212 | 0.02 | 7 | 0.04 ± 0.02 | 54.4 | 1.000 |
|  |  | 2 | 25-212 | 0.04 | 7 |  | 56.6 |  |
|  |  | 3 | 32-212 | 0.06 | 6 |  | 102.6 |  |
|  | FeS | 1 | 17-174 | 0.06 | 8 | 0.04 ± 0.02 | 84.9 | 0.998 |
|  |  | 2 | 17-212 | 0.02 | 8 |  | 77.9 |  |
|  |  | 3 | 25-212 | 0.04 | 8 |  | 84.2 |  |
|  | FeSO_4_ | 1 | 17-46 | 0.33 | 4 | 0.36 ± 0.02 | 89.7 | 0.000 |
|  |  | 2 | 25-46 | 0.37 | 3 |  | 90.8 |  |
|  |  | 3 | 25-46 | 0.37 | 3 |  | 90.0 |  |
|  | Na_2_S | 1 | 10-237 | 0.00 | 10 | 0.01 ± 0.00 | 57.9 | 0.452 |
|  |  | 2 | 25-237 | 0.01 | 9 |  | 76.1 |  |
|  |  | 3 | 32-237 | 0.01 | 6 |  | 68.9 |  |
|  | Na_2_SO_4_ | 1 | 25-87 | 0.05 | 6 | 0.03 ± 0.02 | 93.9 | 1.000 |
|  |  | 2 | 17-258 | 0.03 | 12 |  | 75.8 |  |
|  |  | 3 | 17-258 | 0.02 | 11 |  | 71.7 |  |

^1^Methane production rates were calculated via linear regression of data points between the dates reported in Start and end day columns, where the moles of methane were between 10% and 90% of the maximum methane value. ^2^ Methane yield calculations were conducted based on the oxidation reaction of propionate to methane, resulting in a theoretical conversion coefficient of 1.75 (Singh et al., 2023b).

^2^ P-values from Dunnett’s test for methane production rates (additive vs. control).

**Table 3.** Rates for thermophilic enrichment experiments repeated for assessing the microbial community dynamics (cf. Figure S3, S4).

| Group | Replicate | Start and end day | Rate^1^ (mmol day^-1^) | n (regression points) | Average rate (mmol day^-1^) | Dunnett p-value |
| --- | --- | --- | --- | --- | --- | --- |
| Control | 1 | 12-68 | 0.00 | 9 | 0.01 ± 0.01 |  |
|  | 2 | 5-61 | 0.01 | 9 |  |  |
|  | 3 | 4-53 | 0.01 | 8 |  |  |
| FeCl_2_ | 1 | 20-68 | 0.03 | 8 | 0.03 ± 0.01 | 0.793 |
|  | 2 | 13-61 | 0.03 | 8 |  |  |
|  | 3 | 4-46 | 0.04 | 7 |  |  |
| FeSO_4_ | 1 | 12-68 | 0.09 | 9 | 0.19 ± 0.09 | 0.013 |
|  | 2 | 5-47 | 0.19 | 7 |  |  |
|  | 3 | 11-32 | 0.28 | 4 |  |  |

^1^Propionate oxidation rates were calculated via linear regression of data points between the dates reported in Start and end day columns. These were the dates where propionate depletion fell between 10% and 90% of total propionate depletion, as described by the formula: 0.1 <= (x_start_ – x) / (x_start_-x_end_) < 0.9, where x is the moles of propionate and x_start_/x_end_ is the moles of propionate at first/last propionate data point respectively.

**Table 4.** H_2_S formation and H_2_ levels in triplicate control and triplicate batch assays supplemented with 2.3 mM of FeSO_4_ or Na_2_SO_4_.

| Amendment | Days after incubation | Propionate (mM) | H_2_S | H_2_ (Pa) | Rate (mmol day^-1^)^2^ |
| --- | --- | --- | --- | --- | --- |
| Control | 7 | 27.5 ± 0.3 | 5952 ± 271 | 72 ± 37 | 0.07 ±0.00 |
|  | 14 | 24.0 ± 0.2 | 6132 ± 187 | 61 ± 5 |  |
|  | 17 | 23.3 ± 0.0 | 5914 ± 229 | 23.4 ± 8.1 |  |
| NaSO_4_ | 7 | 27.3 ± 0.2 | 5991 ± 218 | 70 ± 17 | 0.07 ± 0.00 |
|  | 14 | 24.1 ± 0.2 | 6296 ± 108 | 59 ± 6 |  |
|  | 17 | 23.3 ± 0.3 | 6315 ± 252 | 24.8 ± 8.2 |  |
| FeSO_4_ | 7 | 28.1 ± 0.6 | 742 ± 163 | 139 ± 17 | 0.13 ± 0.01 |
|  | 14 | 22.7 ± 0.5 | 2768 ± 1197 | 168 ± 8 |  |
|  | 17 | 19.8 ± 1.0 | 5154 ± 0^1^ | 79.4 ± 11.7 |  |

^1^H_2_S could not be measured for one replicate due to insufficient gas volume.

^2^ Propionate oxidation rates were calculated by linear regression using data from days 7, 14, and 17 post-inoculation, representing the initial phase of oxidation.
